# Hepatitis B Virus Immunization Today

**DOI:** 10.1155/S1064744901000126

**Published:** 2001

**Authors:** Stanley A. Gall

**Affiliations:** Department of Obstetrics and Gynecology University of Louisville School of Medicine Louisville KY 40292 USA


### Immunology update: Hepatitis B virus immunization today

**Stanley A. Gall**

*Department of Obstetrics and Gynecology, University of Louisville School of Medicine, Louisville, KY*

The concept of adult immunization is difficult for many of the general population and of the physician population alike to perceive as important. In reality, the immunization of pediatric-age patients is largely an accepted fact and ongoing process but adults can find many reasons to neglect themselves.

Many physicians are aware that hepatitis B virus (HBV) poses a significant health risk to patients who become infected with this virus. The incidence of HBV infection in the United States is rising despite the availability of effective strategies. Current estimates of the incidence of HBV infection suggest approximately 300 000 new cases annually or an increase of 37% in the last 20 years.

In the US, adolescents and adults account for the majority of new cases. Over 95% of otherwise healthy adults and older children who acquire HBV recover from the infection and suffer no long-lasting effects. In contrast, children infected under the age of 1 year have a 90% chance of developing chronic infection and those under 5 years have a 40–50% chance. Children who become infected with HBV are at a disproportionate risk for the development of major complications including chronic hepatitis with cirrhosis and liver failure, fulminant hepatitis and hepatocellular carcinoma. HBV vaccine is truly the first cancer vaccine.

During the late 1970s, hepatitis B immune globulin (HBIG) was administered to neonates within the first 2 days of life. This strategy prevented neonatal acquisition of the virus and the administration of HBV vaccine given in combination with HBIG not only prevented infection in infancy but conveyed lasting protection in over 95% of treated infants. The current strategy developed by the Centers for Disease Control (CDC) includes recommendations for universal screening of all pregnant women early in pregnancy and repeated screening of women who are at high risk of HBV infection, i.e. intravenous drug users and those with intercurrent sexually transmitted diseases or clinically evident hepatitis. It is strongly recommended that the first dose of HBV vaccine should be given in the hospital prior to discharge. Data from Table 1 indicate that initial protection is present with just one dose. The recommended hepatitis B vaccine schedule is seen in Table 2.

A number of hospitals and health plans have backed away from initiation of HBV vaccination in the newborn nursery. This is a tragic turn of events in the effort to immunize all persons. Each physician must examine his/her own hospital's

**Table 1** Protection available after each dose of hepatitis B vaccine. Data from Margolis HS, Alter MJ, Hadler SC. *Semin Liver Dis* 1991;11:84–92[1]

| *Dose number* | *Infants* | *Teens and adults* |
|---|---|---|
| 1 | 16–40% | 20–30% |
| 2 | 80–95% | 75–80% |
| 3 | 98–100% | 90–95% |

Correspondence to: Stanley A. Gall, MD, Department of Obstetrics and Gynecology, University of Louisville School of Medicine, Louisville, KY 40292

**Table 2** Recommended hepatitis B (HBV) vaccination timings for infants. Engerix-B® (SmithKline Beecham, Rixensart, Belgium) and Recombivax HB® (Merck, Whitehouse Station, NJ) are the vaccine products available for use in the United States. Engerix-B and Recombivax HB single-antigen HBV vaccines are used for the birth dose and can also be used for all the remaining doses recommended for infants and children

| | *When to administer HBIG and HBV vaccine to your infant patients* | | | |
|---|---|---|---|---|
| *Patient history* | *HBIG* | *HBV dose 1* | *HBV dose 2* | *HBV dose 3* |
| Infant born to HbsAg-positive mother | Give within 12 hours of birth | Give within 12 hours of birth | Give at 1–2 months of age | Give at 6 months of age |
| Preterm infant born to HbsAg-positive mother | Give within 12 hours of birth | Give dose 1 within 12 hours of birth. If infant weighs less than 2 kg at birth, repeat dose 1 when infant weighs 2 kg or is 2 months of age | Give 1–2 months after the previous dose | Give at 6 months of age |
| Infant born to HbsAg-negative mother and at high risk of early childhood infection* | No | Preferably give dose 1 at birth but give no later than 2 months of age | Give 1–4 months of age | Give at 6 months of age or no later than 12 months of age |
| Infant born to mother not tested for HbsAg** and mother is later found to be HbsAg positive | Give ASAP before 7 days of age | Give within 12 hours of birth | Give at 1–2 months of age | Give at 6 months of age |
| Infant born to mother not tested for HbsAg** and mother is later found to be HbsAg negative | No | Give within 12 hours of birth | Give at 1–2 months of age | Give at 6–18 months of age |
| Preterm infant born to HbsAg-negative mother | No | Give when infant weighs 2 kg or at 2 months old | Give at 1–4 months of age | Give at 6–18 months of age. Infants at high risk of infection need dose 3 no later than 12 months of age |
| Infant born to HbsAg-negative mother and at low risk of early childhood infection | No | Give at birth–2 months of age | Give at 1–4 months of age | Give at 6–18 months of age |

*Infants born to HbsAg-negative mothers who are at risk of early childhood HBV infection include infants whose mothers belong to populations and groups from areas of moderate and high endemicity for HBV infection. Areas of high endemicity (≥ 8% HBV carrier rate) include Africa; Southeast Asia including China, Korea, Indonesia, and the Philippines; the Middle East except Israel; South and Western Pacific Islands; interior Amazon Basin; and certain parts of the Caribbean, i.e. Haiti and the Dominican Republic. Alaska natives are also at high risk. Areas of moderate endemicity (2–7% HBV carrier rate) include South Central and Southwest Asia, Israel, Japan, Eastern and Southern Europe, Russia, and most of Central and South America. Also, any infant who lives in a household with an HBV carrier should be considered at high risk for HBV infection; **mothers should have blood drawn for HbsAg testing as soon as possible; HBIG, hepatitis B immune globulin; ASAP, as soon as possible policy because the policy may have changed without the involved physicians being alerted. We must therefore insist on universal screening of all pregnant women, as well as universal administration of the first HBV vaccine dose in the newborn nursery.

### REFERENCES

1. Margolis HS, Alter MJ, Hadler SC. Hepatitis B: evolving epidemiology and implications for control. *Semin Liver Dis* 1991;11:84–92
